# A machine learning driven nomogram for predicting chronic kidney disease stages 3–5

**DOI:** 10.1038/s41598-023-48815-w

**Published:** 2023-12-07

**Authors:** Samit Kumar Ghosh, Ahsan H. Khandoker

**Affiliations:** Healthcare Engineering Innovation Center (HEIC), Department of Biomedical Engineering, Khalifa University, Abu Dhabi, United Arab Emirates

**Keywords:** Chronic kidney disease, Chronic kidney disease

## Abstract

Chronic kidney disease (CKD) remains one of the most prominent global causes of mortality worldwide, necessitating accurate prediction models for early detection and prevention. In recent years, machine learning (ML) techniques have exhibited promising outcomes across various medical applications. This study introduces a novel ML-driven nomogram approach for early identification of individuals at risk for developing CKD stages 3–5. This retrospective study employed a comprehensive dataset comprised of clinical and laboratory variables from a large cohort of diagnosed CKD patients. Advanced ML algorithms, including feature selection and regression models, were applied to build a predictive model. Among 467 participants, 11.56% developed CKD stages 3–5 over a 9-year follow-up. Several factors, such as age, gender, medical history, and laboratory results, independently exhibited significant associations with CKD (p < 0.05) and were utilized to create a risk function. The Linear regression (LR)-based model achieved an impressive R-score (coefficient of determination) of 0.954079, while the support vector machine (SVM) achieved a slightly lower value. An LR-based nomogram was developed to facilitate the process of risk identification and management. The ML-driven nomogram demonstrated superior performance when compared to traditional prediction models, showcasing its potential as a valuable clinical tool for the early detection and prevention of CKD. Further studies should focus on refining the model and validating its performance in diverse populations.

## Introduction

Chronic kidney disease (CKD) is an age-related, dangerous, and progressive pathological condition that affects the reduction in kidney function^1–3^. It occurs when the kidneys are damaged and unable to effectively filter waste products from the blood. Over time, the condition may progress to end-stage renal disease (ESRD), where the kidneys lose their ability to perform their essential functions, and patients require kidney dialysis or a kidney transplant to survive^4^. Based on findings from a systematic review, it has been found that approximately 11-13% of the world population is affected by CKD, with the majority of cases falling within the stage of three to five. The incidence of CKD increases in direct proportion to the progression of age. This is supported by empirical evidence indicating that around 35% of individuals who are 70 years old or above are impacted by CKD^5^. CKD is associated with a higher susceptibility to cardiovascular disorders (CVD), such as strokes and heart attacks^6^. In the last 20 years, the prevalence of CKD has significant rise, affecting $13.4\%$ of the global population^7^. Majority of the cases are seen between stages 3 and 5 of CKD^5^. Patients diagnosed with CKD are highly susceptible to the development of cardiovascular diseases, which stand as the primary cause of mortality within this population. Accurate prediction of survival is essential for the management of CKD patients at a significant risk of heart diseases, as it can aid in guiding clinical decision-making and improving patient outcomes. The initial phases of CKD are often asymptomatic, which means that patients may not experience any noticeable symptoms until the disease has progressed to a more advanced stage^3^. As a result, early detection and management of CKD are crucial for preventing the disease’s progression to ESRD and reducing the risk of associated complications such as cardiovascular disease, anemia, and bone disease^8^. The diagnostic process of CKD typically involves blood and urine tests to assess kidney function and identify any abnormalities. Treatment may include medications to regulate blood pressure and blood sugar levels, dietary changes, and lifestyle modifications such as quitting smoking and increasing physical activity^9^. The causes of CKD can vary, but some common risk factors include hypertension, blood pressure, diabetes mellitus, cholesterol levels, smoking, obesity, and a family history of kidney disease^10–13^. Survival forecasting in patients with CKD has traditionally relied on clinical factors such as age, sex, coexisting medical conditions, and laboratory values. However, these factors may not accurately predict survival in all CKD patients, especially those with complex medical histories and multiple comorbidities. With the advent of machine learning algorithms and big data analytics, there is an opportunity to develop more accurate and personalized survival forecasting models for CKD patients^14, 15^. In this study, we conducted an analysis on a dataset consisting of 467 patients released by Al-Shamsi et al.^7^ in 2018. In their original study, the authors used multivariate Cox proportional hazard analysis to find the independent risk factors (older age, history of smoking, history of coronary heart disease, and history of diabetes mellitus) associated with developing CKD stages 3–5. In 2021, following the previous study, Davide et al.^16^ conducted an analysis on the identical dataset. They focused on developing a machine learning approach that could effectively classify the progression of serious CKD and identify the key variables within the dataset. Through a feature ranking analysis, they determined that age, creatinine, and eGFR were the most significant clinical characteristics when the temporal component was absent, whereas hypertension, smoking, and diabetes played a crucial role when considering the year factor. Although the two studies^7, 16^ mentioned above presented interesting results and identified distinct risk factors associated with different stages of CKD, the existing literature lacks robust nomograms specifically designed to predict the risk of incident CKD in high-risk populations of CVD^17^. This study aims to fill this gap by developing a novel nomogram specifically designed for this particular population. The current nomogram serves as a straightforward and dependable tool for stratifying the risk of CKD among populations with a high risk of CVD. Utilizing a risk prediction tool to identify individuals at a higher risk of developing incident CKD can improve primary care for this condition. However, the primary healthcare system encounters several challenges, including a shortage of medical personnel, inadequate government funding, and excessive workloads. To address these issues, it is feasible, convenient, and widely accepted to construct a CKD risk prediction model using conventional data within the medical system, alongside improving chronic disease management techniques. Its purpose is to assist physicians in identifying individuals who are at risk and promptly implementing targeted prevention strategies.

## Materials and methods

### Dataset collection and subject information

**Figure 1:**
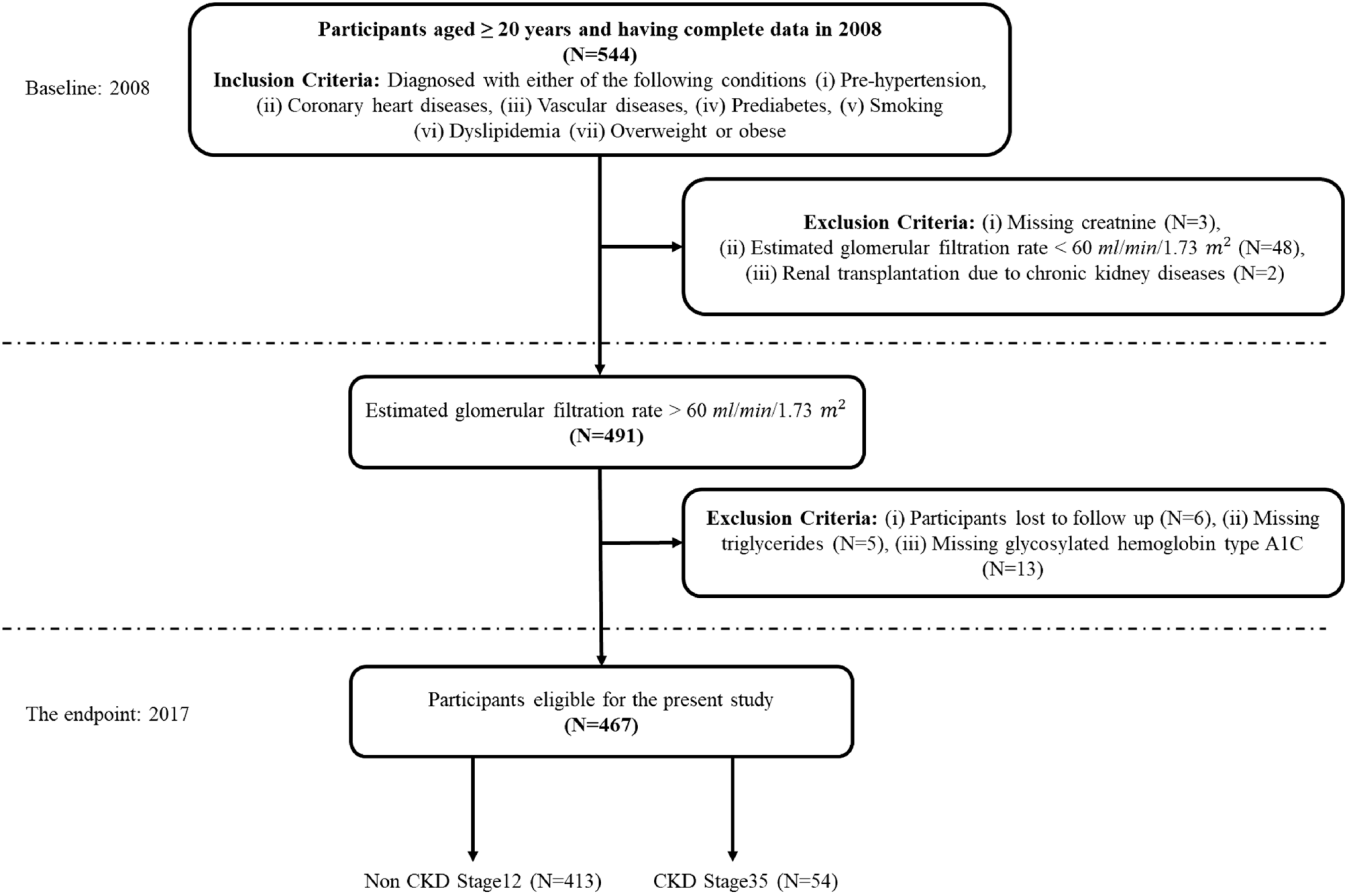
Flow diagram of study design and participants selection.

The present investigation employed a dataset obtained from^7^, which included health records of 544 patients collected from Tawam Hospital located in Al-Ain city, Abu Dhabi, United Arab Emirates (UAE) between January 1, 2008, and December 31, 2008. Figure 1 shows the flow diagram of the study design and patient selection process.

A total of 467 patients were included according to the inclusion and exclusion criteria. Out of which, 234 were female patients and 233 were male patients, aged 23–89 years. Due to the retrospective nature of the study, the need for informed consent was waived by the Tawam Hospital and UAE University Research Ethics Board, which approved the study protocol under Application No. IRR536/17. The study was performed in accordance with the Declaration of Helsinki. All the patients were UAE citizens over the age of 20 and diagnosed with one or more of the following conditions: coronary heart disease (CHD), pre-hypertension, diabetes mellitus (DM) or prediabetes, vascular diseases, dyslipidemia, smoking, or being overweight or obese. The data collected includes the age of the patients ($\le 49$, 50–60, and $\ge 65$), sex (female, male), smoking status (no, yes), obesity (no, yes), total cholesterol (TC), triglycerides (TG), estimated glomerular filtration rate (eGFR), glycosylated hemoglobin type A1C (HbA1C), systolic blood pressure (SBP), diastolic blood pressure (DBP), body mass index (BMI), and serum creatinine (Scr) of the patients. The study also includes disease parameters such as CHD (no, yes), diabetes mellitus (no, yes), hypertension (HTN) (no, yes), dyslipidemia (no, yes), and vascular diseases (no, yes), angiotensin-converting enzyme (ACE) inhibitors and angiotensin II receptor blockers (ARBs) use (no, yes). The category within the parentheses in the definition mentioned above serves as the reference group. Patients were recorded as having CHD if they had evidence of a coronary event, a coronary revascularization operation, or a cardiologist-determined diagnosis. Similarly, patients were categorized as having vascular disease based on specific criteria. These criteria included a documented history of cerebrovascular accident or transient ischemic stroke, a documented history of peripheral arterial disease, or the occurrence of revascularization for peripheral vascular disease. The exclusion criteria of this study were as follows: (i) eGFR less than 60 mL/min/1.73; (ii) patients with incomplete clinical data; (iii) the period of time during which the patient’s follow-up was lost. All dataset attributes refer to the patients’ initial visits in January 2008, except for the time-year variables and EventCKD35 (binary variables 0 and 1). The duration of the follow-up ended in June 2017. The binary variables 0 and 1 indicate that the patients are in CKD stages 1 or 2, and 3, 4, or 5, respectively. During the follow-up period, 54 patients (11.56%) with CKD stages 3–5 were identified in the entire cohort. In the context of this study, ‘time’ refers to the duration of the follow-up period subsequent to patients’ diagnosis and initiation of treatment, which is quantified in terms of survival months. In the sample of 54 patients, the average duration of follow-up was found to be 50 months, with the minimum observed follow-up period being 3 months.

### Diagnostic criteria

The diagnostic criteria for CKD stages 3–5 were defined based on the eGFR and kidney damage, which can be assessed through various diagnostic tests and clinical evaluations. The Kidney Disease Improving Global Outcomes (KDIGO) was used to categorize patients with CKD into two groups: normal (eGFR is $\ge 60$ mL/min/1.73), and CKD stages 3–5 (eGFR is $\le 60$ mL/min/1.73)^18^. The CKD epidemiology collaboration (CKD-EPI) creatinine equation was used to determine eGFR, as per the definition given below^19^:

1 $$\begin{aligned}&\text {eGFR}=141\times \textit{min}(\text {SCr}/\kappa ,1)^{\alpha }\times \textit{max}(\text {SCr}/\kappa ,1)^{-1.209}\times 0.993^{\text {Age}}\nonumber \\&\times \left( 1.018~ \text {if `female'} \right) \times \left( 1.159~ \text {if for `African descent'} \right) \end{aligned}$$

where $\text {SCr}$ denoted seram creatinine measured in $\mu \text {mol}/\text {L}$, age is expressed in years, $\kappa$ is a constant of 0.9 for ‘males’ and 0.7 for ‘females’, $\alpha$ is a constant of $-0.411$ for ‘males’ and $-0.329$ for ‘females’, *‘min’* represents the *‘minimum’* value of $\text {SCr}/\kappa$ or 1, and *‘max’* represents the *‘maximum’* value of $\text {SCr}/\kappa$ or 1^19–21^. A factor of 1.0 was assigned for ethnicity due to the absence of African-descent subjects in this study. The BMI ranges used for identifying individuals as overweight and obese are 25–29.9 kg/$\text {m}^{2}$ and $\ge 30$ kg/$\text {m}^{2}$, respectively. According to^22^, HTN was described as SBP over 140 mmHg, DBP over 90 mmHg, or taking medicine to treat high blood pressure. Diagnostic standards for dyslipidemia included serum TC values of $\ge 6.21$ mmol/L, serum TG levels of $\ge 2.26$ mmol/L, or the use of lipid-lowering drugs^23^. The reference ranges for creatinine were 58-96 $\mu$ mol/L for females and 53–115 mol/L for males^7^. Patients were considered to have a positive smoking history if they reported either current or past tobacco smoking. The definition of prediabetes and DM followed the guidelines set by the American Diabetes Association (ADA)^24^.

### Model estimation and selection

To analyze the data, first, the non-parametric Kaplan–Meier (KM) estimator was used to measure the amount of time spent in follow-up and visualize the survival curves. Then, a semi-parametric Cox proportional hazard regression model was employed to describe the impact of the variables on the survival outcome. These methods are briefly detailed here.

#### Kaplan–Meier method

The KM method is a non-parametric modeling approach established by Kaplan & Meier in 1958 that predicts survival probability based on observed survival^25^. The general formula for determining the survival probability ${\hat{S}}(t)$ at time $t_{i}$ is as follows:

2 $$\begin{aligned} {\hat{S}}(t)=\prod _{t_{i}\le t}\frac{n_{i}-d_{i}}{n_{i}}=\prod _{t_{i}\le t}\left( 1-\frac{d_{i}}{n_{i}} \right) \end{aligned}$$

where $t_{1},t_{2},\cdots ,t_{n}$ are the ordered unique event timings, and $n_{i}$ is the total number of patients that were ‘at risk’ prior to time $t_i$. The variable $d_{i}$ represents the count of instances that have occurred at time $t_{i}$. The estimated probability is a step function that begins with a horizontal line at a survival probability of 1 (when survival probability is $100\%$) and then steps down to zero as survival probability drops. The KM estimates model is used to perform an analysis of the survival probability. The survival time, measured in months, was the primary dependent variable. Follow-up time can be interpreted as a time to event (TTE), where the event would be CKD stages 1–2 or CKD stages 3–5. The non-parametric KM method has a significant drawback: it cannot represent survival probability with a smooth function, rendering it unable to make predictions. On the other hand, parametric models such as the exponential and weibull distribution models can overcome this limitation^26^. They serve as a logical progression from the KM method, bridging the gap and greatly improve understanding of survival analysis. Besides, in cases where parametric models are appropriate, they are more exact, more effective, and more informative than KM. The KM estimation curve fits with exponential and weibull distributions by considering statistical measures such as the AIC (Akaike Information Criterion) and maximum log-likelihood. A model with a smaller AIC value is a better fit, while a model with a higher (maximum) log-likelihood is a good fit. After running the initial analysis, it was seen that the weibull distribution has a larger loglikelihood of − 259.78 and the smallest AIC of 523.56 compared to exponential model estimates (loglikelihood: − 265.49, AIC: 532.98). So, weibull is a superior fit for the model because it follows the statistical preference of maximizing log-likelihood while minimizing AIC for fitting the model and making predictions.

**Figure 2:**
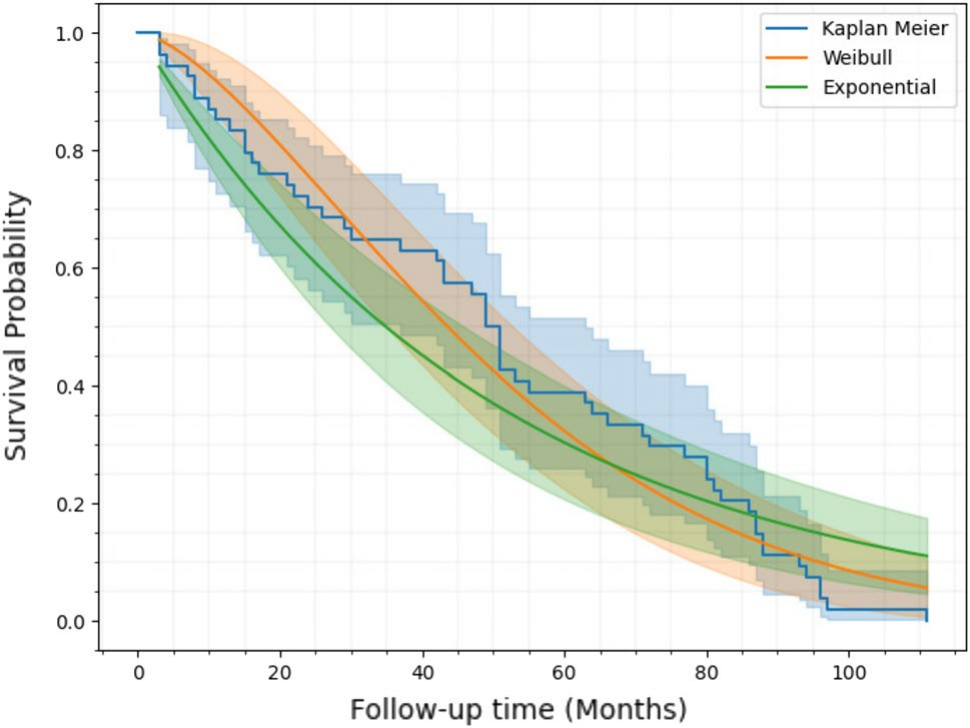
Survival curves for different models: Kaplan–Meier estimator, Weibull distribution, exponential distribution.

**Figure 3:**
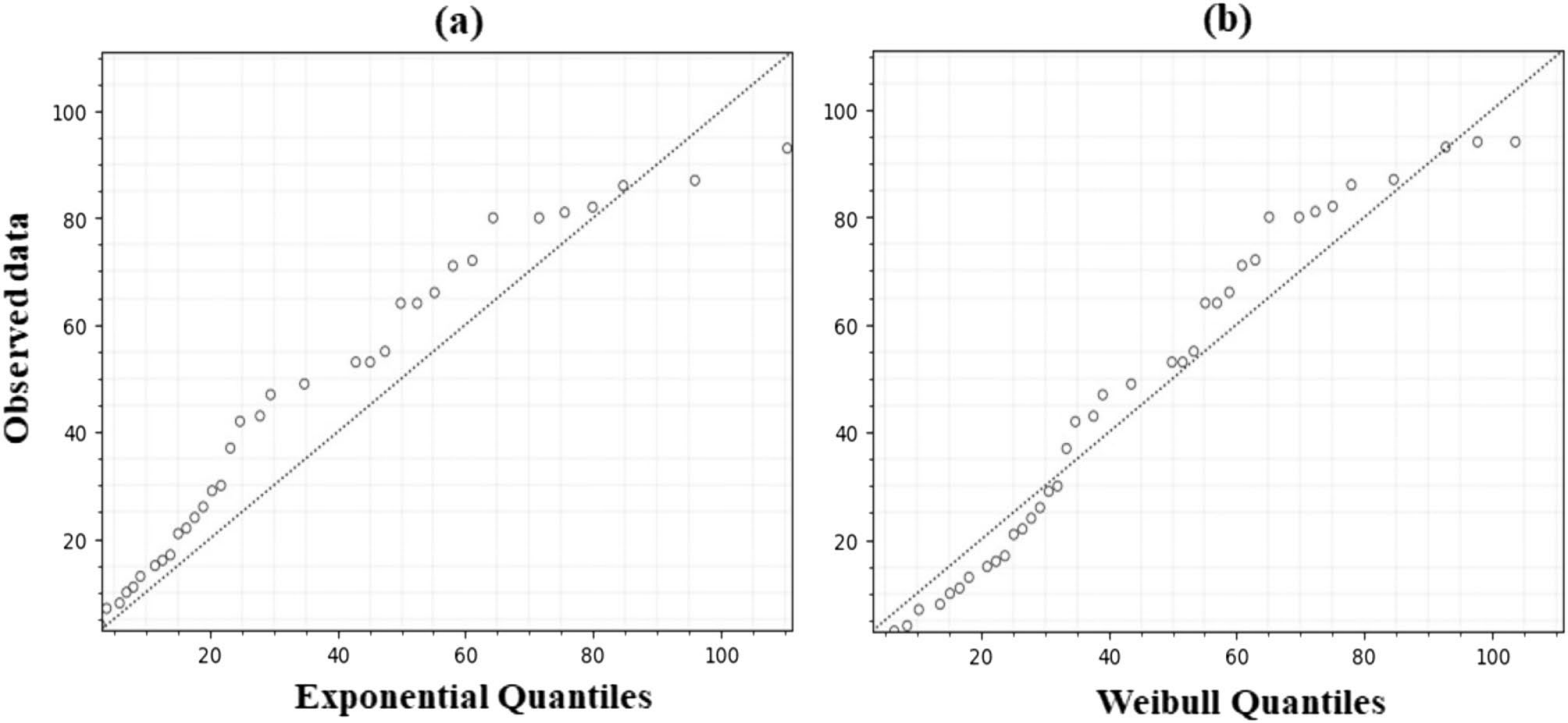
Q–Q plot for (**a**) exponential distribution, (**b**) Weibull distribution.

Figure 2 shows the KM plots for the survival function of CKD patients in stages 3–5 and the visual distribution of both models. The Python programming language (version 3.10.12) and the “lifelines” package were used to estimate the KM curve^27^. It displays the time period (follow-up months) on the *x*-axis and survival probabilities on the *y*-axis. A notable disparity was observed with regards to patient survival. The exponential distribution survival plot, depicted by the green curve (Fig. 2), exhibits a slight deviation from the KM survival plot represented by the blue curve, whereas the orange plot aligns with it. The smooth rate of decrease observed in the described approach effectively characterizes the survival probability, surpassing the step-wise nature of the KM method, which experiences abrupt drops in probability only following an event while maintaining constant probabilities between events. In order to determine which model provides the best fit, a comparison of the quantile–quantile (Q–Q) plot (as shown in Fig. 3) is used to check the clustering of observations along a slope line^28^.

The Q–Q plot determines which distribution provides a better fit to the KM estimation survival curve. The distribution whose Q–Q plot aligns more closely with a straight line indicates a better fit to the data. If the points deviate significantly from a straight line, it indicates that the data does not fit the chosen distribution well. From Fig. 3, it can be observed that the weibull distribution is a good fit for the model as most of the data points (observed data) seem to be clustered along the slope line. Hence, we can use the weibull distribution model to predict other features affecting CKD patients in stages 3–5; this will help us determine which features are most strongly associated with patients’ survival.

#### Cox proportional hazard model

The Cox proportional hazard model is a semi-parametric method that can be used to analyze survival-time outcomes, also known as time-to-event outcomes, based on one or more predictors^29^. The model demonstrates features of a general regression analysis, which enables the evaluation of different levels of a factor’s influence on survival time while accounting for other factors. Its functionality is highly similar to that of the logistic regression model, but instead of predicting a binary outcome, it focuses on time-to-event data. The computation of the regression coefficient enables the determination of the relative risk that is linked to the corresponding factor. The logistic regression model is designed to handle only qualitative variables as the dependent variable, such as the outcome of a case (the end event), without incorporating the duration of survival time. The Cox hazard-based model utilizes survival time and event occurrence as its dependent variables. The Cox proportional hazards model is presented in the following form of an equation^30^:

3 $$\begin{aligned} h(t,{\textbf {X}})=h_{0}(t)e^{g({\textbf {X}})} \end{aligned}$$

where, *t* represents the time, and ${\textbf {X}}$ indicates a number of contributing factors. The relative risk function, denoted as $g({\textbf {X}})=\beta ^{\text {T}}{} {\textbf {X}}$, is solely dependent on the *p* explanatory variables ${\textbf {X}}=x_{1},x_{2},\cdots , x_{p}$ and the regression parameter $\beta$. The exponential values of $e^{\beta }$ are called hazard ratios (HR). A positive value of $\beta _{i}$ or a HR greater than one indicates that an increase in the $i^{{\textrm{th}}}$ covariate leads to an increase in the event hazard, resulting in a decrease in the survival length. In other words, a covariate with an HR over 1 is one that is positively correlated with the likelihood of an occurrence and hence negatively correlated with the duration of survival.

## Results and discussion

In this study, a total of 467 participants with eGFR greater than or equal to 60 mL/min/1.73 $\text {m}^{2}$ was considered during every 3-month follow-up period from baseline visit to June, 30 2017. After a period of follow-up, a total of 54 new cases (male: 34; female: 20) of CKD stages 3–5 were identified. There are 233 males and 234 females in this study, and their ages range between 23 and 89 years old (Table 1).

**Table 1:** Explanation, measurement units, and intervals of each feature of the dataset.

Feature	Explanation	Measurement	Range
Gender	Female or male	Boolean	0, 1
Age	Age of the patient	Years	[23, 24, $\ldots$, 89]
History diabetes	If the patient has diabetes	Boolean	0, 1
History CHD	If the patient has coronary heart diseases	Boolean	0, 1
History vascular	If the patient has vascular diseases	Boolean	0, 1
History smoking	If the patient smokes	Boolean	0, 1
History HTN	If the patient has history of hypertension	Boolean	0, 1
History DLD	If the patient has history of dyslipidemia	Boolean	0, 1
History Obesity	If the patient has history of obesity	Boolean	0, 1
DLD meds	If the patient has taken dyslipidemia medications	Boolean	0, 1
DM meds	If the patient has taken diabetes medications	Boolean	0, 1
HTN meds	If the patient has taken hypertension medications	Boolean	0, 1
ACEIARB	If the patient has taken ACEI or ARB	Boolean	0, 1
Cholesterol	Level of cholesterol	mmol/L	[2.23, 2.40, $\ldots$, 9.30]
Triglycerides	Level of triglycerides	mmol/L	[0.18, 0.22, $\ldots$, 6.24]
HgbA1C	Level of glycosylated hemoglobin type A1C	%	[3.90, 4.10, $\ldots$, 18.10]
Creatinine	Level of creatinine in the blood	$\upmu$mol/L	[6, 27, $\ldots$, 123]
eGFR	Estimated glomerular filtration rate	mL/min/1.73$\text {m}^{2}$	[60, 60.4, $\ldots$, 242.6]
SBP	Systolic blood pressure	mmHG	[92, 95, $\ldots$, 177]
DBP	Diastolic blood pressure	mmHG	[41, 45, $\ldots$, 112]
BMI	Body mass index of the patient	kg/$\text {m}^{2}$	[16, 17, $\ldots$, 57]
Time	Follow-up period	Months	[3, 4,$\ldots$, 111]
(Target) CKD Event	Moderate or extreme CKD during the follow-up period	Boolean	0, 1

*ACEI* angiotensin-converting enzyme inhibitors, *ARB* angiotensin II receptor blobkers, *kg* kilogram, *mmol* millimoles, *mmHg* millimetre of mercury.

The oldest male was 89 years old, and the oldest female was 79 years old. Among 233 males, 199 were in CKD stages 1–2 and 34 were in CKD stages 3–5. Similarly, among 234 females, 214 were in CKD stages 1–2 and 20 were in CKD stages 3–5. The dataset contains a total of 23 features (numerical and categorical) that report demographic, biochemical, and clinical information about the CKD patients. The categorical features include the gender of the patient. Additionally, personal history factors are considered, such as diabetes history, CHD history, vascular disease history, smoking history, HTN history, DLD history, and obesity history. Furthermore, specific-disease medicines, namely DLD medications, diabetes medications, HTN medications, and inhibitors (angiotensin-converting enzyme inhibitors or angiotensin II receptor blockers), are represented as binary values (0, 1). A descriptive statistical analysis was done using a mean ± standard deviation (SD) with an unpaired, two-tailed *t*-test for continuous variables and a frequency distribution for categorical variables (using the Chi-squared test) to find out about the patients and their medical conditions. The statistical quantitative description of the categorical and numerical features are described in Tables 2 and 3, respectively. It has been observed from the Table 2 that CKD group subjects (stages 3–5) have a higher history of dyslipidemia (83.33% vs 63.68%), obesity (57.41% vs 51.33%), DLD-Meds (77.78% vs 53.75%), HTN (85.19% vs 60.29%), diabetes (87.04% vs 39.95%), CHD (31.48% vs 6.78%), vascular diseases (11.11% vs 5.08%), smoking (24.07% vs 13.56%), diabetes mellitus (75.93% vs 28.57%), and ACEIARB (77.78% vs 41.89%) than non-CKD group subjects (stages 1–2). The differences in baseline characteristics of the CKD and non-CKD groups (CKD stages 1–2) of subjects in this study are presented in Table 3. The mean age of the non-CKD group ($52.65\pm 13.71$ years) was significantly lower than that of CKD group ($62.70\pm 9.21$ years). The levels of triglycerides (TG), glycosylated hemoglobin type A1C (HbA1C), serum creatinine (SCr), and systolic blood pressure (SBP) in the CKD group were significantly higher as compared to the non-CKD group, but the estimated glomerular filtration rate (eGFR), cholesterol, diastolic blood pressure (DBP), and body mass index (BMI) were lower. The data are expressed as the median, mean, and standard deviation. A *p*-value less than 0.05 was considered statistically significant. It has been observed from Table 3 that the *p*-value of the covariates such as age, cholesterol, triglycerides, HgbA1C, creatinine, eGFR, SBP, and time follow-up is less than 0.05, and this indicates that these variables had a significant impact on the CKD stage 3–5. The other covariates have no significant influence.

**Table 2:** Statistical and quantitative description of the category features.

	Total patients (N = 467)		CKD patients stages 1–2 (N = 413)		CKD patients stages 3–5 (N = 54)	
Categorical feature	Number	Percentage (%)	Number	Percentage (%)	Number	Percentage (%)
Gender (0: female)	234	50.10	214	51.82	20	37.04
Gender (1: male)	233	49.90	199	48.18	34	62.96
Diabetes (0: false)	255	54.60	248	60.05	7	12.96
Diabetes (1: true)	212	45.40	165	39.95	47	87.04
CHD (0: false)	422	90.36	385	93.22	37	68.52
CHD (1: true)	45	9.64	28	6.78	17	31.48
Vascular diseases (0: false)	440	94.22	392	94.92	48	88.89
Vascular diseases (1: true)	27	5.78	21	5.08	6	11.11
Smoking (0: false)	398	85.22	357	86.44	41	75.93
Smoking (1: true)	69	14.78	56	13.56	13	24.07
HTN (0: false)	142	30.41	138	33.41	4	7.41
HTN (1: true)	325	69.59	275	66.59	50	92.59
DLD (0: false)	159	34.05	150	36.32	9	16.67
DLD (1: true)	308	65.95	263	63.68	45	83.33
Obesity (0: false)	224	47.97	201	48.67	23	42.59
Obesity (1: true)	243	52.03	212	51.33	31	57.41
DLD meds (0: false)	203	43.47	191	46.25	12	22.22
DLD meds (1: true)	264	56.53	222	53.75	42	77.78
DM meds (0: false)	308	65.95	295	71.43	13	24.07
DM meds (1: true)	159	34.05	118	28.57	41	75.93
HTN meds (0: false)	172	36.83	164	39.71	8	14.81
HTN meds (1: true)	295	63.17	249	60.29	46	85.19
ACEIARB (0: false)	252	53.96	240	58.11	12	22.22
ACEIARB (1: true)	215	46.04	173	41.89	42	77.78

**Table 3:** Statistical and quantitative description of the numerical features.

	Total patients (N = 467)			CKD patients stage 1–2 (N = 413)			CKD patients stage 3–5 (N = 54)			*p*-value
Numerical feature	Median	Mean	SD	Median	Mean	SD	Median	Mean	SD	
Age	55.00	53.81	13.64	53.00	52.65	13.71	62.00	62.70	9.21	$<0.001$
Cholesterol	5.00	4.98	1.10	5.00	5.04	1.09	4.40	4.54	1.11	0.002
Triglycerides	1.10	1.32	0.80	1.08	1.29	0.80	1.36	1.53	0.72	0.043
HgbA1C	6.10	6.61	1.71	6.00	6.38	1.42	7.50	8.30	2.57	$<0.001$
Creatinine	66.00	67.75	17.81	64.00	65.78	16.93	84.00	82.85	17.26	$<0.001$
eGFR	97.70	97.66	18.40	99.60	100.08	17.67	77.95	79.13	12.39	$<0.001$
SBP	131.00	131.62	15.56	130.00	130.88	15.12	139.00	137.30	17.73	0.004
DBP	77.00	77.04	10.71	77.00	77.29	10.53	74.50	75.09	11.91	0.156
BMI	30	30.41	6.19	30.00	30.43	6.22	30.50	30.28	6.01	0.868
Time	93.00	84.67	24.22	95.00	89.17	19.00	50.00	50.22	31.36	$<0.001$

In this study, we employed the KM survival curve fitting approach in combination with the weibull distribution to analyze and model the survival data. The aim was to determine the “decay rate” with respect to the follow-up time period, which was used as the dependent variable for subsequent regression models. The initial step involved fitting the KM survival curve using the weibull distribution. We produced an accurate representation of the survival data by computing the two parameters of the Weibull distribution, $\gamma$ (shape parameter) and $\lambda$ (scaling parameter). This allowed us to calculate the shape and scale of the survival curve, providing valuable insights into the underlying survival trends. After obtaining the parameters $\gamma = 1.53$ and $\lambda = 55.35$, we determined the decay rate for the follow-up time. This result was used as the dependent variable in our regression models. We employed two regression techniques: Support Vector Machine (SVM)^31^ and Linear Regression (LR)^32^ to investigate the relationship between the decay rate and other relevant features. To identify the most influential features, a feature ranking process was performed, which led to the selection of the top 11 predictors. Using the “SelectKBest” class in Python 3.10.12 with scikit-learn (version: 1.2.2), we specifically employed feature ranking to pinpoint the top 10 most relevant features. This method allowed us to extract features with the highest scores, as determined by statistical tests, underscoring their significance in our analysis and leveraging the chi-squared scoring function for feature selection. These top 11 features were carefully chosen to enhance both the predictive accuracy of our models and the interpretability of the results. Subsequently, these selected features served as the inputs for our regression models, contributing to a more comprehensive understanding of the relationship between these features and the decay rate. For our regression analysis, we adopted a data partitioning strategy, allocating 70% of the data for training the model and reserving the remaining 30% for testing and validation purposes. To assess the performance of the regression analyses, different metrics are used, namely R-score (R-squared), mean squared error (MSE), root mean squared error (RMSE), and mean absolute error (MAE). The MAE is a matric used to measure the average squared difference between the original and predicted values obtained by averaging the absolute differences over the entire dataset. It gives an indication of how close the predictions are to the actual values. The MSE is a measure of the average squared difference between the original values and the predicted values. It is calculated by squaring the average difference over the dataset. RMSE is generated from MSE and provides the error rate of the prediction model. It is evaluated by taking the square root of MSE. RMSE is a popular metric since it provides a measure of the average magnitude of the prediction errors. It helps to understand the magnitude of the errors in the predictions. R-squared, alternatively referred to as the coefficient of determination, It indicates the goodness of fit of the model by measuring how well the predicted values align with the original values. R-squared can be interpreted as the percentage of variability in the dependent variable that is explained by the independent variables. The value of R-squared ranges between 0 and 1, with a higher R-squared value indicating a better fit and 1 representing a perfect fit. The scores obtained from both the SVM and Linear Regression models were tabulated and compared in Table 4 in order to select the best prediction model.

**Table 4:** Comparison of prediction models using MSE, RMSE, MAE and $\text {R}^{2}$.

Model	MSE	RMSE	MAE	$\text {R}^{2}$
SVM regression	0.005628	0.075018	0.069163	0.934005
Linear regression	0.004834	0.069526	0.051722	0.954079

**Figure 4:**
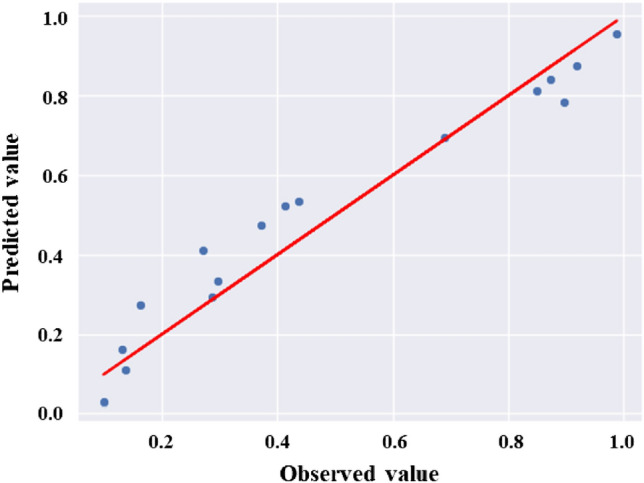
Actual vs. prediction plot for linear regression.

Based on the comparison results provided in Table 4, it is evident that linear models exhibit superior performance on this dataset. In order to obtain an optimal regression model, it is desirable to minimize the error, aiming for a value close to zero, while simultaneously maximizing the variability of the target variable explained by the features, striving for a value close to one. Interestingly, the results indicated that the Linear Regression model outperformed the SVM model, demonstrating better predictive accuracy for the used dataset. Therefore, we consider linear regression models having the lowest RMSE (0.069526) and the highest $\text {R}^{2}$ (0.954079) as the final prediction models. The performance of the linear regression model was assessed by comparing the actual observed values with the predicted values. Figure 4 presents the ‘Actual vs. Prediction’ plot, where each data point represents an observation in the dataset. The *x*-axis represents the observed values of the dependent variable, while the *y*-axis corresponds to the predicted values based on the regression model.

It can be observed from the plot that the majority of the data points align along a diagonal line, indicating a reasonably strong linear relationship between the predicted and actual values. This alignment indicates that the model has successfully captured the underlying trends in the data. However, it is evident that a small number of data points deviate from the diagonal line, indicating a certain level of discrepancy or inaccuracy in the predictions. These deviations could be attributed to various factors, such as measurement errors or unaccounted variables that influence the dependent variable. The ‘Actual vs. Prediction’ plot demonstrates the satisfactory performance of the linear regression model in capturing the inherent relationship between the predictors and the dependent variable. The model’s capability to predict values that fall within a reasonable range of the observed values suggests its reliability for making accurate predictions and extracting meaningful insights from the data. We have conducted a thorough evaluation of our predictive model using the five-fold cross-validation approach. This approach involves partitioning the dataset into five subsets, training the model on four subsets, and evaluating its performance on the remaining subset. This process is repeated five times, ensuring that each subset serves as the validation set exactly once. Table 5 provides a comparison of the cross-validation-based model performance metrics. By utilizing the cross-validation approach, we have ensured a robust assessment of its performance. The results from this comprehensive evaluation confirm that our predictive model is reliable and demonstrate its effectiveness.

**Table 5:** Cross-validation-based model performance metrics comparison.

Model	Metrics	Training set	Validation set
SVM regression	MSE	0.0060	0.0079
	RMSE	0.0777	0.0891
	MAE	0.0719	0.0860
	R^2^	0.9219	0.9370
Linear regression	MSE	0.0024	0.0026
	RMSE	0.0495	0.0510
	MAE	0.0378	0.0394
	R^2^	0.9682	0.9793

To estimate the impact of various covariates on CKD stage 3–5, a semi-parametric Cox hazard model was fitted using the ‘lifelines’ module in Python 3.10.12; the obtained results are presented in Table 6.

**Table 6:** Significance of variables under Cox regression analysis and highlighted estimated coefficients those are significant.

Covariate	$\beta$	$e^{\beta }$	Se$(\beta )$	95% CI for $e^{\beta }$	*z*	*p*
Gender	0.1774	1.1941	2.0613	[0.0210, 67.8699]	0.0860	0.9314
Age	0.0319	1.0324	0.0581	[0.9212, 1.1569]	0.5491	0.5829
History diabetes	− 0.4991	0.6070	0.9680	[0.0910, 4.0478]	− 0.5156	0.6061
History CHD	1.4012	**4.0603**	0.5933	[**1.2690, 12.9908**]	2.3615	**0.0181**
History Vascular	− 0.8333	0.4345	0.9830	[0.0632, 2.9842]	− 0.8477	0.3965
History Smoking	− 0.7466	0.4739	0.6170	[0.1414, 1.5888]	− 1.2100	0.2262
History HTN	− 0.9810	0.3749	0.9988	[0.0529, 2.6556]	− 0.9821	0.3260
History DLD	1.8573	6.4068	1.0272	[0.8554, 47.9809]	1.8080	0.0706
History Obesity	− 0.0040	0.9959	0.8631	[0.1834, 5.4073]	− 0.0047	0.9962
DLD Medications	− 2.5255	**0.0800**	1.1418	[**0.0085, 0.7500**]	− 2.2117	**0.0269**
DM Medications	1.2012	3.3243	0.6503	[0.9292, 11.8926]	1.8471	0.0647
HTN Medications	0.7190	2.0525	1.0752	[0.2495, 16.8855]	0.6687	0.5036
ACEIARB	0.2679	1.3072	0.8125	[0.2658, 6.4276]	0.3297	0.7415
Cholesterol	0.2922	1.3393	0.2270	[0.8582, 2.0901]	1.2869	0.1981
Triglycerides	− 0.2401	0.7865	0.2992	[0.4375, 1.4139]	− 0.8023	0.4223
HgbA1C	− 0.0480	0.9531	0.1010	[0.7818, 1.1618]	− 0.4753	0.6345
Creatnine	0.0203	1.0205	0.1052	[0.8303, 1.2544]	0.1937	0.8464
eGFR	− 0.0069	0.9931	0.1090	[0.8020, 1.2297]	− 0.0633	0.9495
SBP	0.0348	**1.0354**	0.0164	[**1.0025, 1.0694**]	2.1125	**0.0346**
DBP	− 0.0399	0.9608	0.0240	[0.9166, 1.0071]	− 1.6636	0.0961
BMI	0.0174	1.0175	0.0625	[0.9001, 1.1503]	0.2786	0.7805

**Figure 5:**
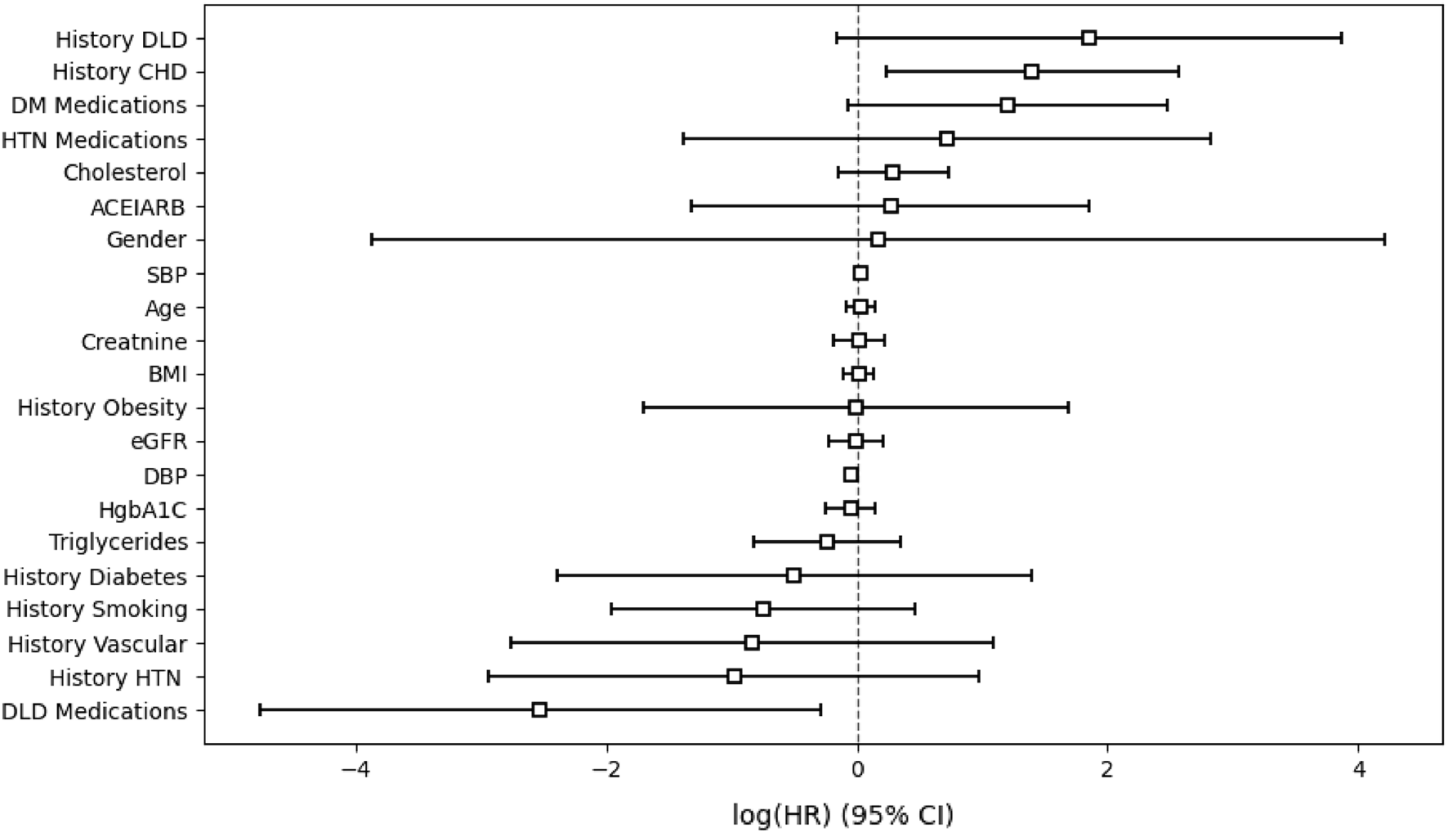
Cox proportional hazard model variable ranking based on log(HR).

The HR and corresponding *p* values for each of the twenty one variable sets are listed in this table. The HR was used to evaluate the relative risk of a variable. If the HR is greater than one, it implies that the variable is positively connected with the likelihood of CKD stage 3–5 and negatively correlated with survival time. On the other hand, if the HR is less than one, it shows that the correlation is in the other direction. It has been observed from Table 6 that the *p*-value of the covariates such as history of CHD, DLD medications and SBP is less than 0.05, and this indicates that these variables had a significant impact on the CKD stage 3–5. The other covariates have no significant influence. The *p*-value for history of CHD is $<0.05$ and the HR is 4.0603 indicating a strong relationship between the patients’ history of CHD and CKD stage 3–5. The variable ranking based on CKD stage 3–5 is illustrated in Fig. 5.

The figure provides a forest plot reporting the HR and the $95\%$ confidence intervals (CI) of the HR for each covariate included in the Cox proportional hazards model. Only history of CHD, DLD medications, and SBP were found to be significant with 0.05 cutoff. It is evident from looking at the figure that history of CHD have a positive influence on survival time while DLD medications have a negative influence on the survival time. The concordance index, or C-index^33^, provides a measure of the discriminative ability of the KM estimate and the Cox Proportional Hazards model in our study. Remarkably, the KM estimate achieved a perfect C-index of 1.0, signifying its impeccable ability to distinguish between different outcomes and accurately order survival times within our dataset. In contrast, the Cox Proportional Hazards model yielded a C-index of 0.7510, indicating a substantial but not flawless discriminatory power. This comparison suggests that the KM estimate outperforms the Cox model in terms of discrimination, demonstrating an unparalleled capacity to precisely predict survival outcomes within our specific context. The KM estimate and the Cox Proportional Hazards model are both important tools in survival analysis, but they serve different purposes and have distinct advantages. Here are some advantages of the KM estimate over the Cox Proportional Hazards model: (i) KM estimates provide a non-parametric way to estimate survival curves. They make no assumptions about the underlying hazard function, which can be advantageous when the assumptions of the Cox model do not hold, (ii) KM curves are easily interpretable and can be plotted to visualize survival probabilities over time for different groups or categories. This makes them valuable for descriptive and exploratory analysis, (iii) KM analysis is relatively simple and does not involve the complexities of modeling covariates. It’s a suitable choice when you want to focus solely on estimating and comparing survival probabilities between groups, (iv) KM is the method of choice when the primary goal is to examine and describe the time-to-event data without modeling covariates. It is particularly useful for studying event occurrence in clinical trials and observational studies. However, it is important to note that while the KM estimate has these advantages, it is limited in its ability to model the impact of covariates on survival time and does not provide HRs. For such analyses, the Cox proportional hazards model may be more appropriate. Following the selection of the superior regression model, we extracted the coefficients and intercept values from the model. These coefficients and intercepts were crucial in constructing a nomogram. A nomogram is a graphical representation that provides a simple and intuitive tool for predicting outcomes based on the regression model. It consists of four lines: the point line, the line for the risk factor, the line for the probability, and the line for the total number of points. The process of constructing these lines has been previously explained^34, 35^. The point line is built by assigning values ranging from 0 to 100. The linear predictor ($\text {LP}_{mn}$) value is determined based on a coefficient derived from a fitted regression model. If the independent attributes $\text {X}$ is a categorical with *n* categories, and ($n-1$) dummy variables are generated. The formula for $\text {LP}_{mn}$ is as follows:

4 $$\begin{aligned} \text {LP}_{mn}=\beta _{mn}\times \text {X}_{mn} \end{aligned}$$

Using this formula, $\text {PointS}_{mn}$ are calculated for each risk category and aligned to the respective risk factor lines. The calculation for $\text {PointS}_{mn}$ is as follows:

5 $$\begin{aligned} \text {PointS}_{mn}=\frac{\text {LP}_{mn}-\min _{n}{(\text {LP}_{mn})} }{\max _{n}{(\text {LP}_{*n})}-\min _{n}{(\text {LP}_{*n})}} \times 100 \end{aligned}$$

where $\beta _{mn}$ represents the regression coefficient value for the *n*th category of the *m*th risk factor. $\text {LP}_{*n}$ indicates the $\text {LP}$ value of the risk factor with the largest estimated range of attribute values. The probability line indicates the probability value associated with a given total point, which spans the range from 0 to 1. The total point line is derived by cumulatively summing up the $\text {PointS}_{mn}$ values.

6 $$\begin{aligned} \text {Total Points} = \sum _{mn}\text {PointS}_{mn}=\sum _{mn}\frac{\text {LP}_{mn}-\min _{n}{(\text {LP}_{mn})} }{\max _{n}{(\text {LP}_{*n})}-\min _{n}{(\text {LP}_{*n})}} \times 100 \end{aligned}$$

The Logistic Regression model is represented by the expression $\sum _{mn}\text {LP}_{mn}$. The total number of points corresponding to each value of the probability line can be determined by substituting this equation into the previous expression.

7 $$\begin{aligned} \text {Total Points} =\sum _{mn}\frac{ln\left( \frac{\text {P}(\text {Y}=1|\text {X}=x)}{1-\text {P}(\text {Y}=1|\text {X}=x)} \right) -\alpha -\sum _{mn}\min _{n}{(\text {LP}_{mn})} }{\max _{n}{(\text {LP}_{*n})}-\min _{n}{(\text {LP}_{*n})}} \times 100 \end{aligned}$$

**Figure 6:**
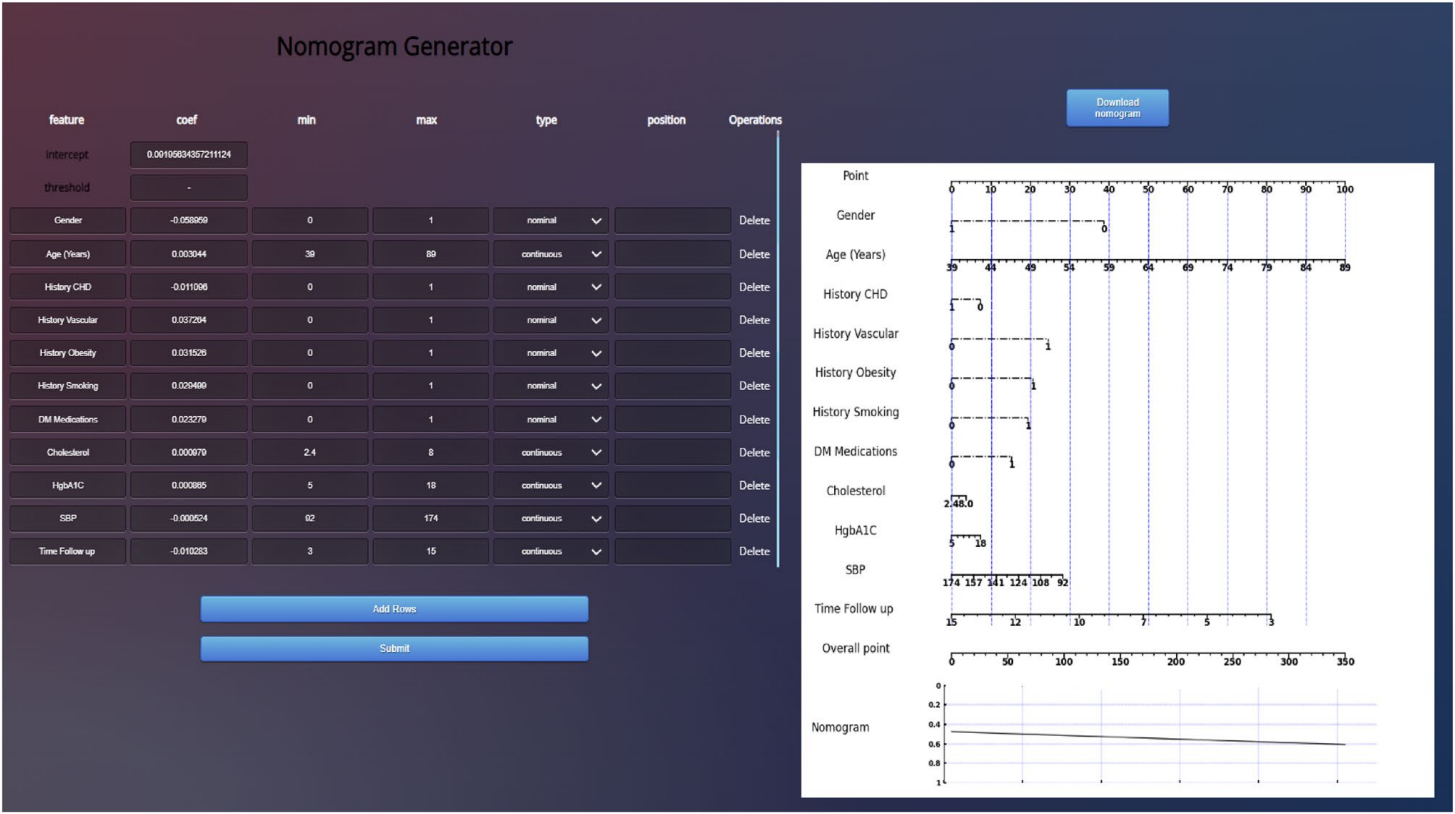
Generate the nomogram with online generator.

In this equation, the value on the probability line, $\text {P}(\text {Y}=1|\text {X}=x)$ is substituted to construct the total point line. By utilizing the coefficients and intercept value ($\alpha$), a nomogram can be developed as shown in Fig. 6 to aid in clinical decision-making and risk assessment^34^.

**Figure 7:**
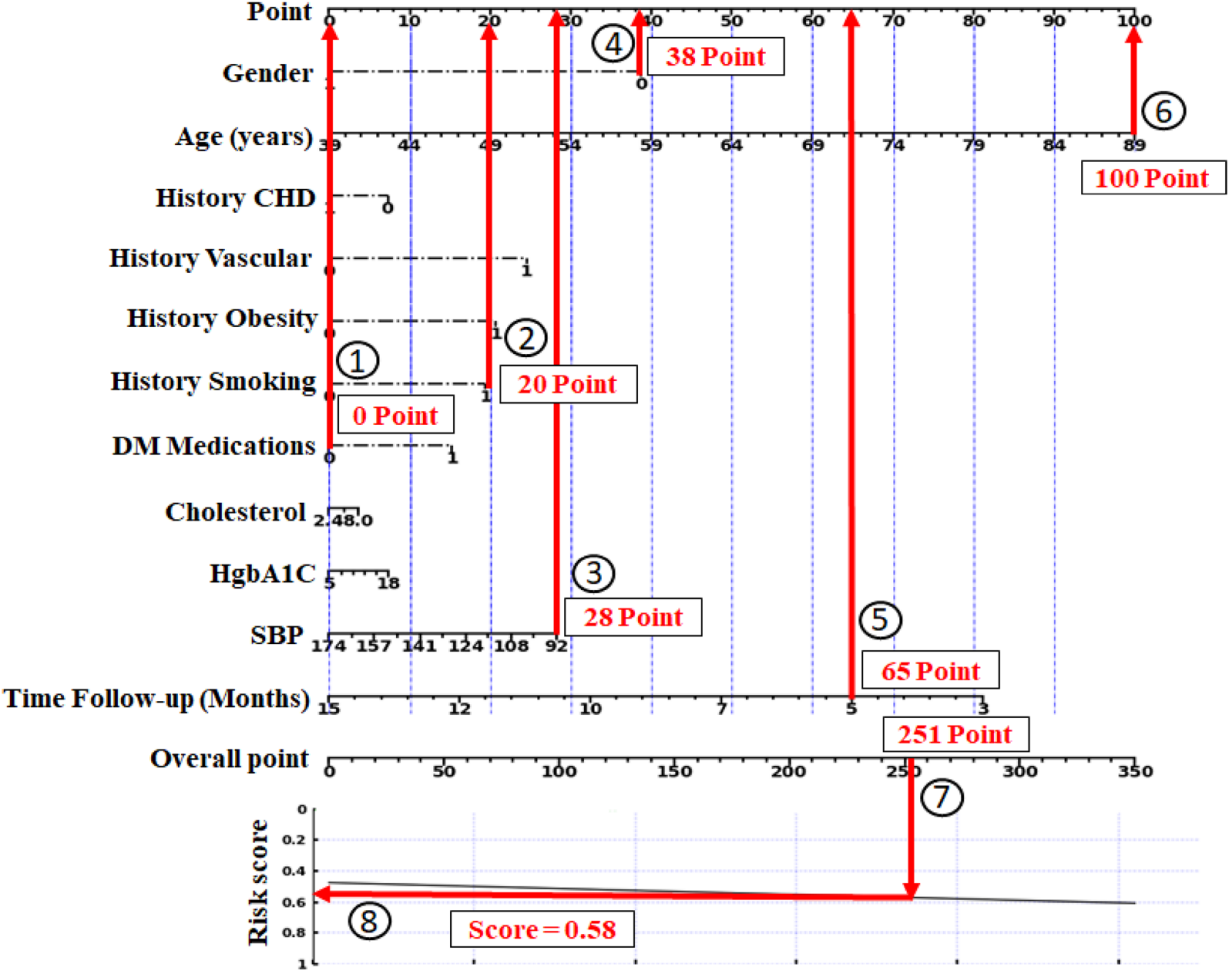
An example of Nomogram results for CKD stages 3–5 to predict risk score.

To predict the risk of CKD stages 3–5 for a patient with the following values: gender = 0, age = 89, history of smoking = 1, DM medications = 1, SBP = 92, and time follow-up = 5 months, each value is assigned to its respective points as illustrated in Fig. 7.

**Figure 8:**
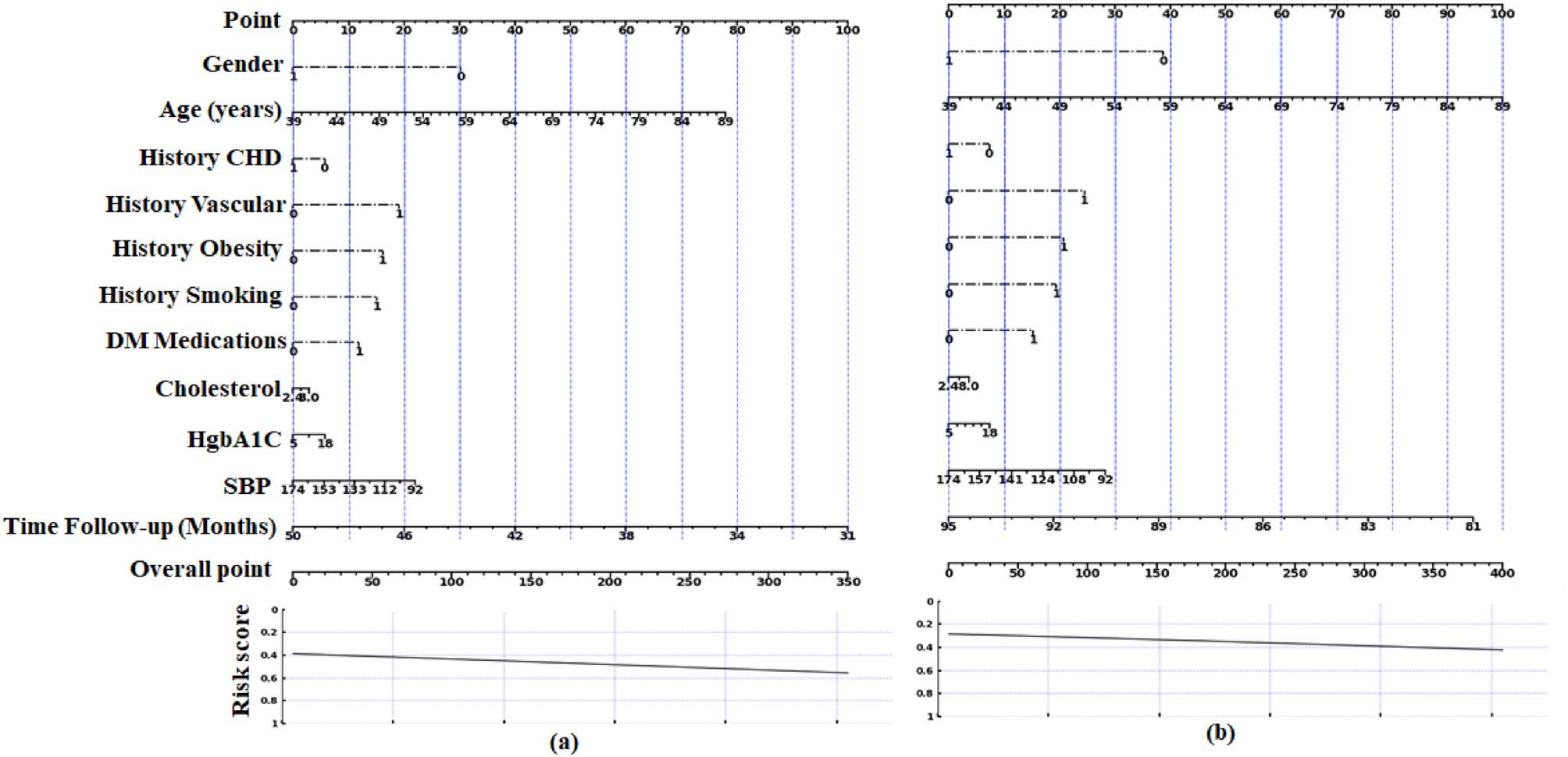
Nomogram results predicting the risk scores: (**a**) during follow-up months 31–50; (**b**) during follow-up months 81–95.

The resulting point values obtained are as follows: 38, 100, 20, 0, 28, and 65. These numbers are then summed to get an overall point value of 251, which may be used to assess the risk of CKD stages 3 to 5 by consulting the nomogram’s given curve. Using these data, we may estimate that this patient has a 0.58% chance of developing CKD stages 3–5. This example demonstrates the practical applications of nomograms to predict clinical outcomes. Figure 8 shows the nomogram results indicating the risk scores based on the established logistic regression model during the follow-up periods of 31–50 and 81–95 months, respectively.

**Figure 9:**
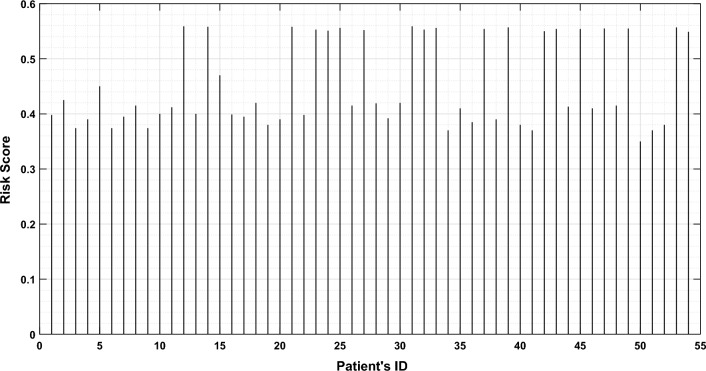
Plot depicting the patient’s ID versus risk score.

Additionally, supplementary Figs. S1, S2, S3, and S4 provided the corresponding results for the follow-up periods of 16–30 months, 51–65 months, 66–80 months, and 96–111 months, respectively. The nomogram assessment considered various factors such as age, gender, medical history, laboratory results, and specific risk factors associated with CKD stages 3–5. By integrating these factors, we have generated personalized risk scores for each patient. These risk scores are visually represented in Fig. 9 and the summary of results is provided in supplementary Table ST1.

The plot depicting the patient’s ID versus risk score for CKD stages 3–5 provides a visual representation of the varying levels of risk associated with individual patients within these stages. The *x*-axis of the plot corresponds to the patient ID, which is a unique identifier assigned to each patient within the dataset. The patient IDs are organized in ascending order, meaning that the patients’ data points will be plotted sequentially along the *x*-axis. The vertical *y*-axis, is used to represent the risk score associated with stages 3–5 of CKD. The risk score is a quantitative measure that evaluates the probability or seriousness of complications associated with CKD. Through an analysis of the plot, one can observe the distribution of risk scores across the patients with CKD stages 3–5. Higher risk scores are typically associated with patients who have a higher probability of developing complications from their kidney disease. Conversely, lower risk scores indicate a lower probability of such events occurring. The plot allows healthcare professionals to visually identify the risk scores of patients with CKD stages 3–5. It can assist in identifying patients who may require closer monitoring, targeted interventions, or specialized care based on their individual risk profiles. Additionally, the plot can provide insights into the overall distribution of risk scores within this specific CKD population, helping to inform future clinical decision-making. The study has several flaws: (i) the small size of the datasets; (ii) since patient mortality was not taken into account in this study, the incidence of CKD may be underestimated; (iii) more information about the patient’s physical features and work history would have helped find other risk factors for cardiovascular diseases; and (iv) if a similar dataset with similar characteristics from a different part of the world had been available, it would have been helpful.

## Conclusion

This study presents a novel machine learning-driven nomogram for predicting CKD stages 3–5. The proposed approach offers an accurate and personalized risk assessment tool with the potential to improve early detection and preventive strategies. The integration of advanced machine learning algorithms and comprehensive patient data contributes to the robustness and reliability of the developed nomogram. This proposed nomogram has great predictive capacity and may have major clinical implications for diagnosing CKD stages 3–5. Future research needs to focus on the integration of additional data sources and validation through prospective studies, fostering the translation of this nomogram into clinical practice, and improving patient outcomes.

### Supplementary Information

Supplementary Information.

## Data Availability

All data relevant to the study are included in the article or uploaded as supplementary information. The datasets utilized and/or examined in the present study can be accessed from the following source: https://figshare.com/articles/dataset/6711155?file=12242270.
