# Author Correction: A machine learning driven nomogram for predicting chronic kidney disease stages 3–5

**DOI:** 10.1038/s41598-024-51817-x

**Published:** 2024-01-15

**Authors:** Samit Kumar Ghosh, Ahsan H. Khandoker

**Affiliations:** Healthcare Engineering Innovation Center (HEIC), Department of Biomedical Engineering, Khalifa University, Abu Dhabi, United Arab Emirates

Correction to: *Scientific Reports* 10.1038/s41598-023-48815-w, published online 07 December 2023

The original version of this Article contained errors, where in two instances the word ‘Nomogram’ was incorrectly misspelt as ‘Monogram’.

As a result, in the Abstract section

“This study introduces a novel ML-driven monogram approach for early identification of individuals at risk for developing CKD stages 3–5.”

now reads

“This study introduces a novel ML-driven nomogram approach for early identification of individuals at risk for developing CKD stages 3–5.”

and

“An LR-based monogram was developed to facilitate the process of risk identification and management.”

now reads

“An LR-based nomogram was developed to facilitate the process of risk identification and management.”

The original Article has been corrected.

