# Supplementary material for: A machine learning driven nomogram for predicting chronic kidney disease stages 3–5
Source: Sci Rep. 2023 Dec 7;13:21613. doi: 10.1038/s41598-023-48815-w (PMC10703939; doi:10.1038/s41598-023-48815-w)
Supplement: Supplementary file 1 — Supplementary Information. [file 41598_2023_48815_MOESM1_ESM.pdf]

Supplementary material

Figure S1. Nomogram results predicted the risk scores during follow-up months 16 to 31.
Figure S2. Nomogram results predicted the risk scores during follow-up months 51 to 65.
Figure S3. Nomogram results predicted the risk scores during follow-up months 66 to 80.
Figure S4. Nomogram results predicted the risk scores during follow-up months 96 to 111.
Table T1. Risk scores for each patient in CKD stages 3-5.

Supplementary Figure S1. Nomogram results predicted the risk scores during follow-up months 16 to 31.

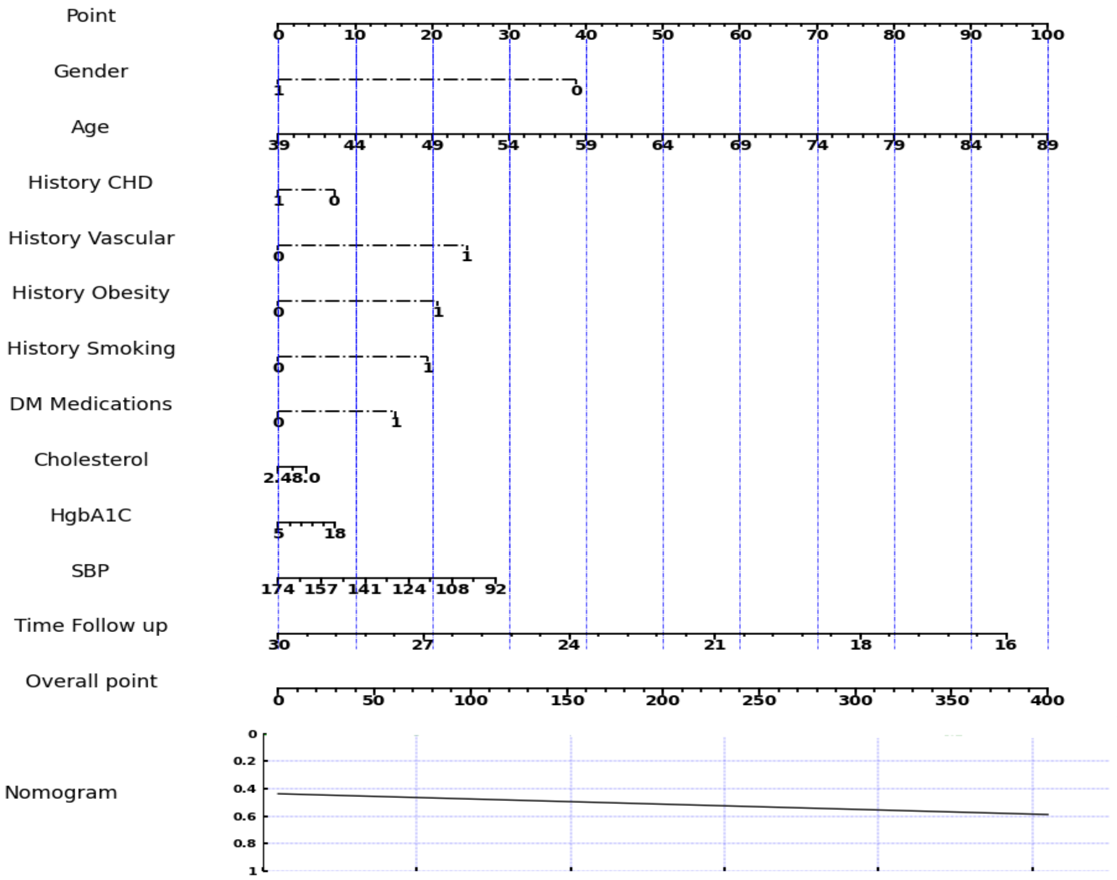

Supplementary Figure S2. Nomogram results predicted the risk scores during follow-up months 51 to 65.

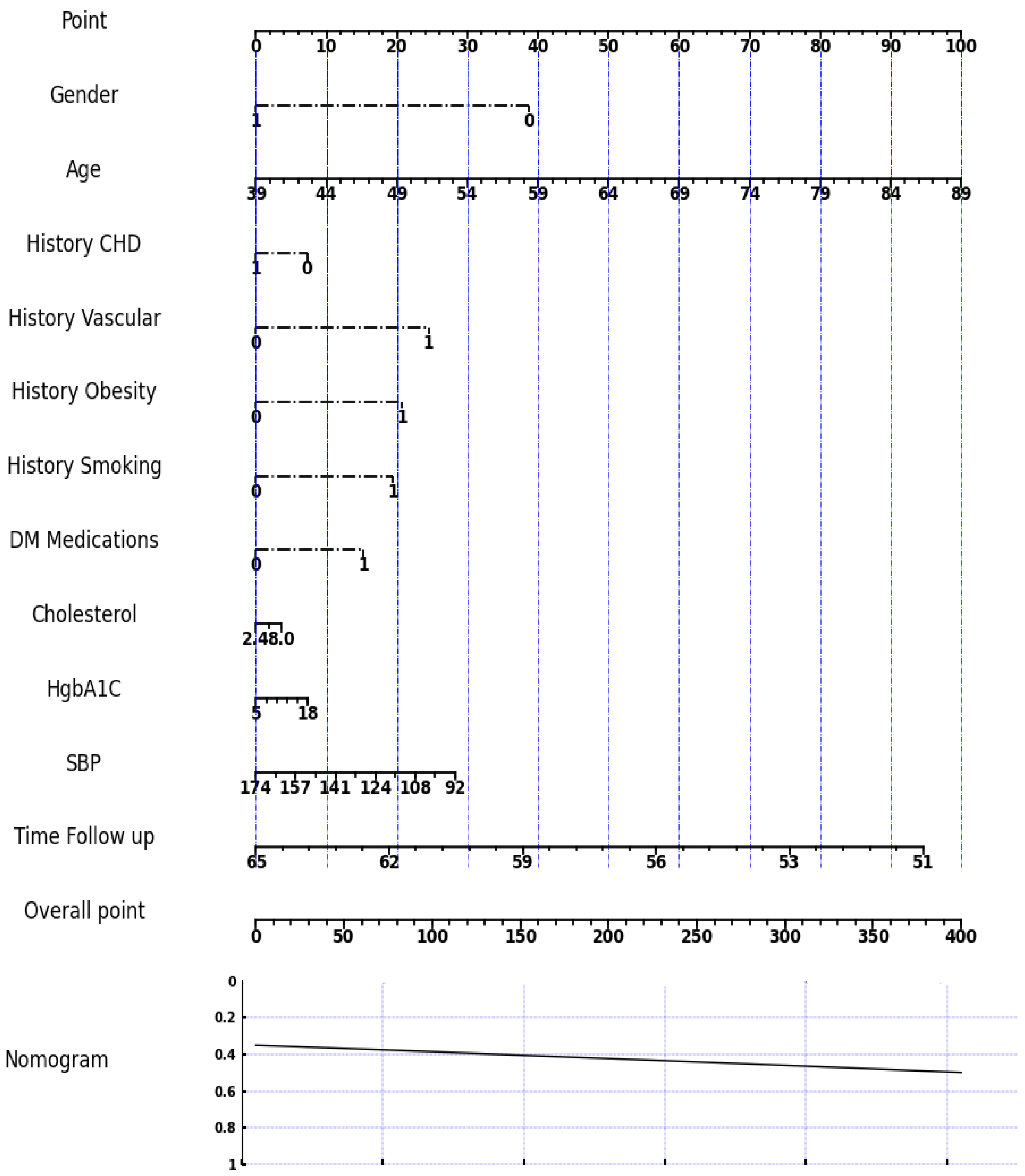

Supplementary Figure S3. Nomogram results predicted the risk scores during follow-up months 66 to 80.

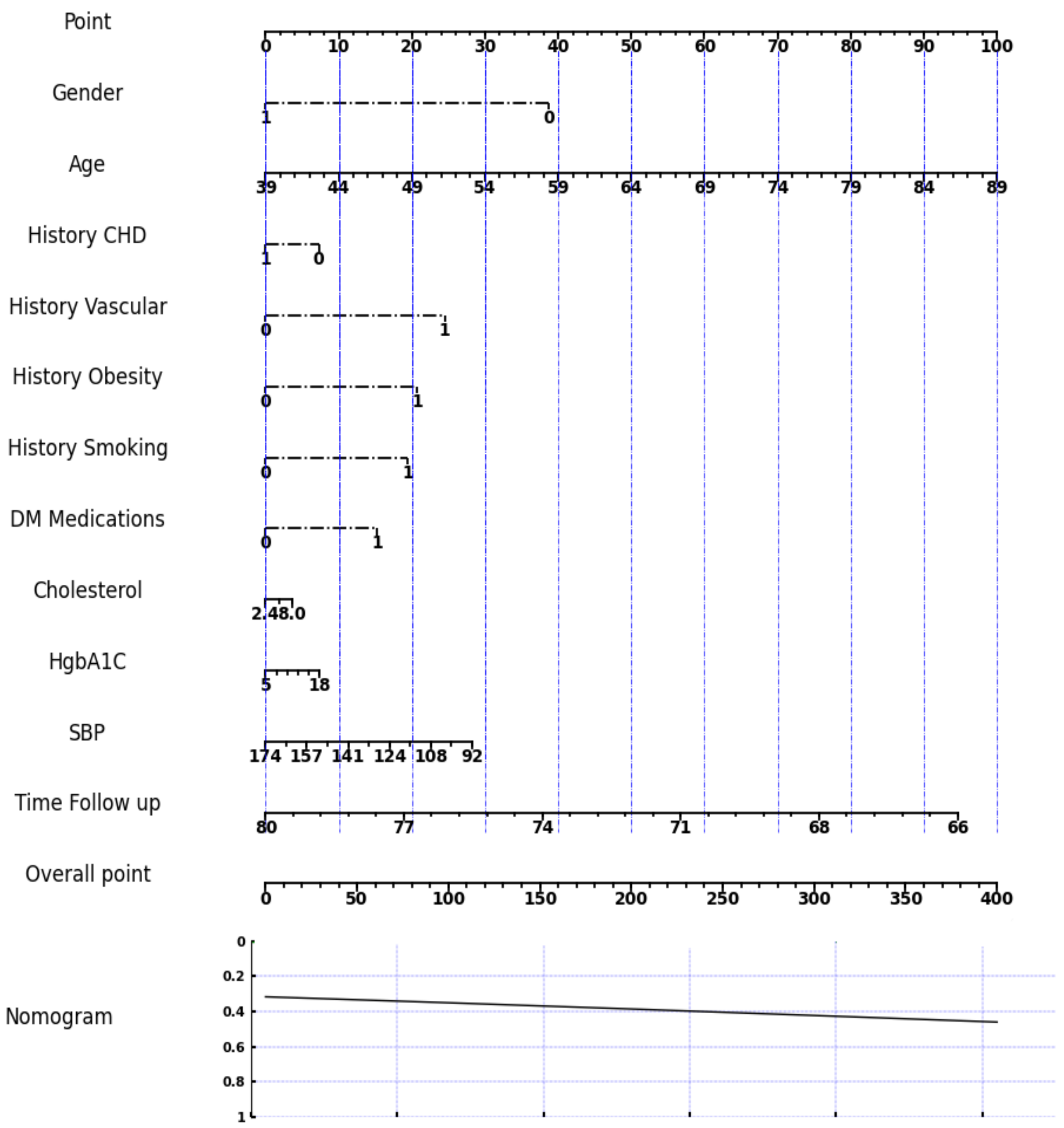

Supplementary Figure S4. Nomogram results predicted the risk scores during follow-up months 96 to 111.

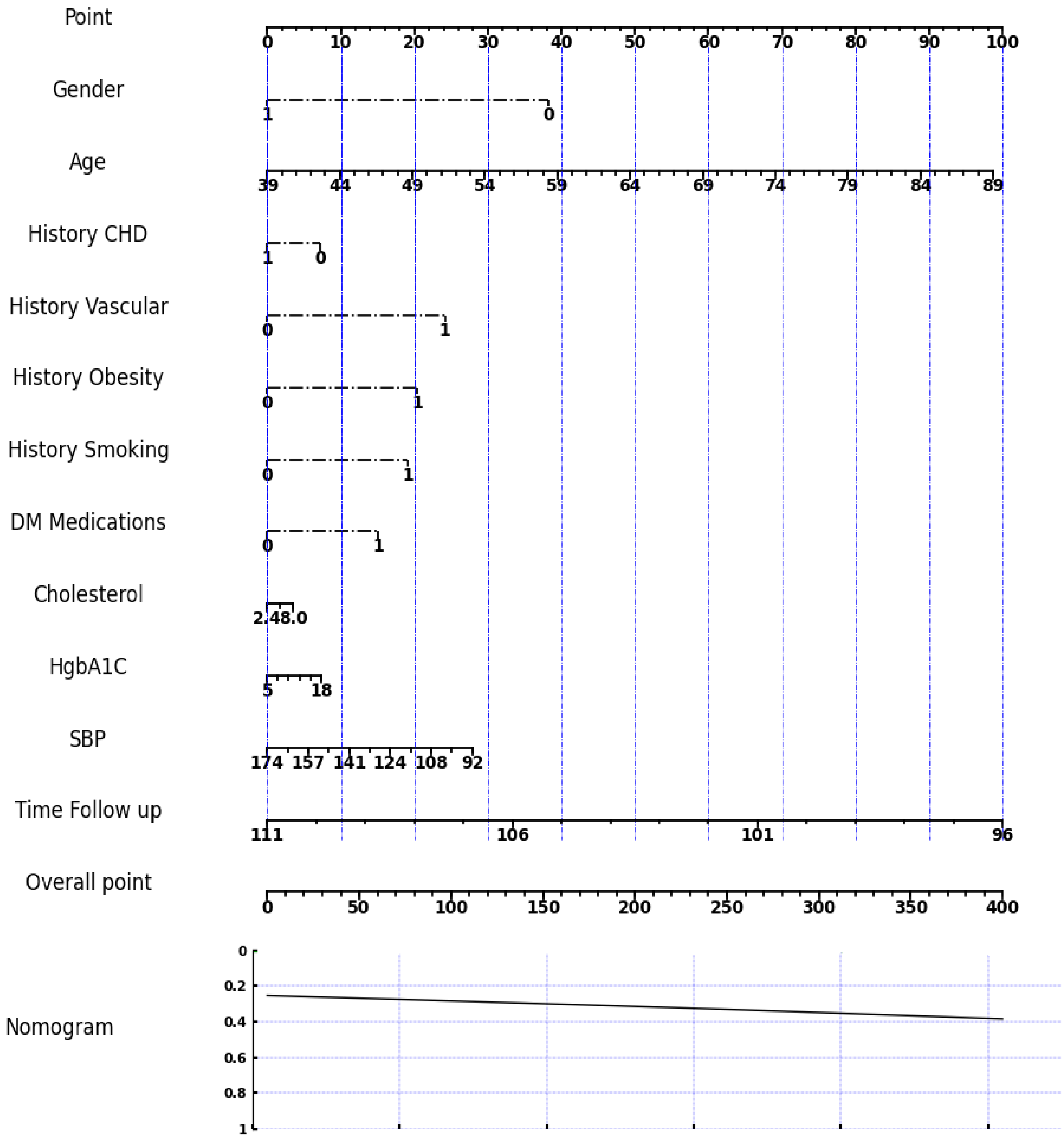

Table ST1. Risk scores for each patient in CKD stages 3-5.

Patient's ID	Gender	Age	Hist CHD	Hist Vas	Hist Obes	Hist Smoke	DM meds	Chol	HgbA1C	SBP	Time To Event	Event_CKD35	Total Point	Risk Score
1	0	79	0	0	1	0	1	5.30	7.10	146	81	1	271	0.398
2	0	69	0	0	1	0	1	4.70	9.60	125	47	1	148	0.425
3	1	72	0	0	1	0	0	5.20	7.30	145	88	1	152	0.374
4	1	69	0	0	0	1	0	5.80	5.60	166	72	1	150	0.390
5	0	61	0	0	0	0	1	4.10	9.60	114	37	1	168	0.450
6	1	57	1	1	0	1	1	3.30	12.90	142	87	1	165	0.374
7	0	76	0	0	1	0	0	3.90	7.60	140	96	1	255	0.395
8	0	54	0	0	1	0	1	5.40	8.30	167	51	1	214	0.415
9	1	55	0	0	0	1	1	3.70	9.40	119	86	1	153	0.374
10	0	66	0	0	1	0	0	4.40	5.08	140	63	1	152	0.400
11	1	76	0	0	1	0	0	5.50	7.00	135	55	1	188	0.412
12	1	61	1	0	1	1	1	4.60	7.50	150	29	1	117	0.559
13	1	56	1	0	0	1	0	3.60	5.70	125	51	1	170	0.400
14	0	55	1	0	1	0	1	3.00	6.87	132	30	1	122	0.558
15	0	68	0	0	1	0	1	5.80	7.10	132	43	1	161	0.470
16	1	56	1	0	0	0	1	4.40	10.90	126	66	1	164	0.399
17	0	54	0	0	1	0	1	5.00	6.20	158	71	1	177	0.395
18	0	66	1	0	0	0	1	2.90	8.50	145	42	1	137	0.420
19	1	64	0	0	1	1	0	4.20	7.60	152	80	1	110	0.380
20	0	51	0	0	1	0	1	4.40	8.37	174	64	1	118	0.390
21	1	56	0	0	1	0	1	4.70	7.30	133	15	1	96	0.558
22	0	55	0	0	1	0	1	5.50	6.80	103	77	1	161	0.398
23	1	71	1	0	0	0	1	4.90	7.50	150	8	1	135	0.553
24	1	73	1	0	1	0	1	3.50	8.00	133	7	1	173	0.551
25	1	79	0	0	0	0	1	4.30	6.50	122	3	1	204	0.556
26	0	57	0	0	1	0	1	5.20	9.60	142	49	1	110	0.415
27	1	60	1	0	1	1	1	3.90	8.50	121	10	1	152	0.552
28	1	62	0	0	0	0	1	4.50	9.00	114	49	1	80	0.419
29	0	54	0	0	1	0	1	5.70	9.10	107	88	1	183	0.392
30	0	65	0	0	1	0	1	3.80	12.70	159	51	1	239	0.420
31	1	60	0	0	0	0	0	5.30	5.30	144	11	1	86	0.559
32	1	65	0	0	1	0	1	2.60	9.60	144	16	1	164	0.553
33	1	55	1	0	1	0	1	4.90	12.38	151	21	1	138	0.556
34	0	61	0	0	0	0	0	4.00	5.60	134	93	1	120	0.370
35	0	69	0	0	1	0	1	4.30	6.50	149	49	1	128	0.410
36	0	57	0	0	1	0	1	4.50	6.10	107	80	1	147	0.385
37	0	64	1	0	1	0	0	4.20	6.60	139	24	1	156	0.554
38	0	53	0	0	1	0	1	7.10	11.00	131	87	1	183	0.390

39	1	42	1	0	1	0	1	3.97	7.90	140	8	1	102	0.557
40	1	66	0	1	0	1	1	3.50	7.10	138	96	1	238	0.380
41	1	62	0	0	1	0	1	4.90	8.15	137	97	1	198	0.370
42	1	66	0	0	0	0	1	4.30	7.00	115	3	1	182	0.550
43	1	39	0	0	0	1	1	8.00	18.10	138	17	1	155	0.554
44	1	89	0	0	0	0	0	2.90	5.97	110	53	1	209	0.413
45	1	61	0	1	0	1	1	4.80	5.83	157	13	1	130	0.554
46	1	55	0	0	1	0	1	6.60	7.10	142	51	1	186	0.410
47	1	75	0	0	0	0	0	5.80	6.20	134	22	1	148	0.555
48	1	67	1	1	0	0	1	5.00	8.80	162	43	1	120	0.415
49	1	66	1	0	0	1	1	2.90	13.50	139	15	1	112	0.555
50	1	66	0	0	1	0	0	3.90	6.80	120	111	1	106	0.350
51	1	55	0	1	0	1	1	6.20	14.90	164	94	1	120	0.370
52	1	62	1	0	0	0	1	2.40	6.70	92	82	1	176	0.380
53	1	58	1	0	1	1	1	3.70	10.30	157	26	1	131	0.557
54	1	76	1	1	0	0	1	4.40	7.43	153	4	1	200	0.549
